# Small Bowel Volvulus as Delayed Presentation of Undiagnosed Crohn’s Disease: A Case Report

**DOI:** 10.5811/cpcem.2021.8.53524

**Published:** 2021-10-05

**Authors:** Minh Thu T. Nguyen, Amir Ali, Ryan P. Bodkin

**Affiliations:** * University of Rochester Medical Center, Department of Internal Medicine, Rochester, New York; † University of Rochester Medical Center, Department of Emergency Medicine, Rochester, New York

**Keywords:** volvulus, small bowel obstruction, mesenteric ischemia, Crohn’s disease, case report

## Abstract

**Introduction:**

Emergency department (ED) visits related to flare-ups of inflammatory bowel disease (IBD) are becoming more prevalent. There are many potentially dangerous complications and sequelae of uncontrolled IBD.

**Case Report:**

We report a case of a middle-aged woman who presented with a few hours of severe abdominal pain, nausea, and vomiting. Given her hemodynamic instability, she was sent urgently for computed tomography, which showed an incomplete small bowel malrotation, mesenteric volvulus, and high-grade small bowel obstruction with evolving ischemia. The patient underwent exploratory laparotomy to resect most of her small intestines. Biopsies later revealed active Crohn’s disease.

**Conclusion:**

Patients with flare-ups of IBD are common in the ED, but very few present with a midgut volvulus later in life. Our case is unique and adds to the literature due to the dramatic consequences of undiagnosed Crohn’s disease in a patient with intermittent symptoms and extensive workup spanning over two decades.

## INTRODUCTION

Abdominal pain is a common complaint in the emergency department (ED). Abdominal pain associated with flare-ups of inflammatory bowel disease (IBD) is an important consideration in the right clinical context. In 2014, 137,946 ED visits in the United States were attributed to IBD, a significant increase from the 90,846 visits in 2006.^1^ With the rising prevalence of IBD-related visits, emergency physicians should become familiar with the dangerous complications of IBD, including fistula formation, intra-abdominal abscesses, bowel perforation, and small bowel obstruction (SBO).^2^ Approximately 3–7% of SBOs are secondary to Crohn’s disease.^3^ Volvulus with concomitant SBO as a consequence of stricturing Crohn’s disease is an even rarer entity, with very few cases reported in the literature.^4^–^6^ We describe a case of acute midgut volvulus as a delayed presentation of undiagnosed Crohn’s disease.

## CASE REPORT

A 38-year-old woman presented to the ED after a few hours of sudden-onset severe abdominal pain, accompanied by bloating, nausea, vomiting, and diaphoresis. She reported having a bowel movement shortly before her symptoms started and had otherwise been in her normal state of health. Upon the first examination, she appeared cold, clammy, and pale, with a moderately distended abdomen that was diffusely tender to touch without peritoneal signs. Initial vitals showed a blood pressure of 90/65 millimeters mercury, respiratory rate 40 breaths per minute, heart rate 93 beats per minute, temperature 34.1º degree Celsius, and oxygen saturation 100% on room air. The patient recalled dealing with intermittent abdominal pain and bloating for the prior two decades without a clear answer despite multiple evaluations. She was previously diagnosed with abdominal migraines, irritable bowel syndrome (IBS), small intestinal bacterial overgrowth, and gluten sensitivity. Prior workup included upper and lower endoscopies, small bowel follow-through, and magnetic resonance enterography. Her most recent imaging demonstrated numerous, dilated segments of small bowel without prominent inflammation. The patient had been scheduled for a video capsule endoscopy a year prior but could not complete the procedure due to loss of insurance and employment during the coronavirus disease 2019 pandemic.

**Image 1:**
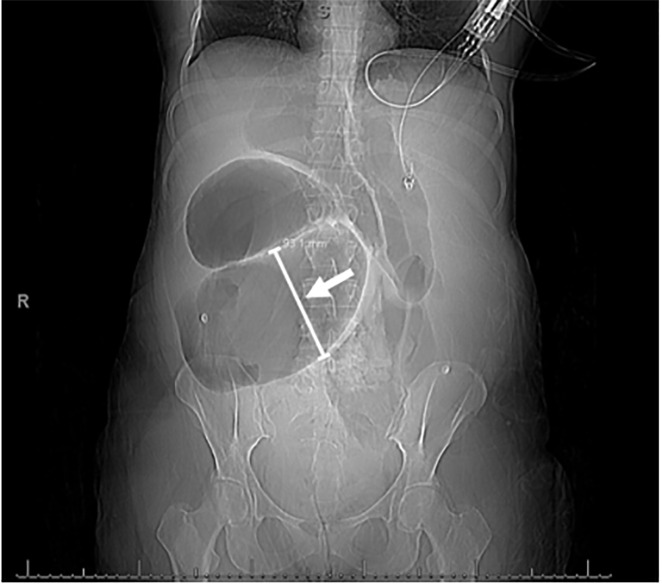
Coronal view of the patient’s scout film prior to computed tomography shows acute midgut volvulus. Loops of proximal small bowel were severely dilated (arrow), measuring 9.31 centimeters in the central anterior abdomen.

**Image 2:**
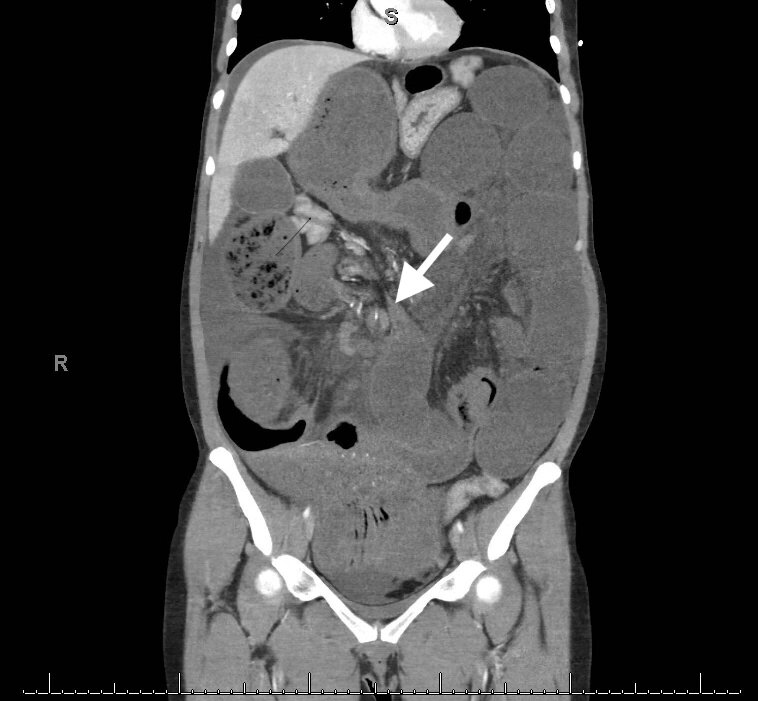
Coronal view of the patient’s computed tomography, showing one of the two transition points (arrow) noted of the high-grade small bowel obstruction located in the right mid-abdomen. There was a small amount of free fluid in the dependent aspects of the abdomen, likely reactive.

Preliminary laboratory data was remarkable for white blood cell count of 11,000 per cubic milliliter (K/uL) (reference range: 4.2 – 9.1 K/uL); hemoglobin of 17.3 milligrams per deciliter (mg/dL) (13.7 – 17.5 mg/dL); C-reactive protein of <3 mg per liter (mg/L) (0 – 8 mg/L); erythrocyte sedimentation rate of 3 millimeters per hour (mm/hr) (0 – 20 mm/hr); lactate of 8.7 millimoles per liter (mmol/L) (0.5 – 2.2 mmol/L); arterial blood gas with pH 7.10 (7.35 – 7.45); and bicarbonate of 16 mmol/L (21 – 26 mmol/L). Given the severity of lactic acidosis and progressive hypotension, aggressive intravenous (IV) crystalloid fluid resuscitation was started, totaling 7 liters. Urgent computed tomography (CT) with IV contrast revealed incomplete small bowel malrotation, mesenteric volvulus at the level of small bowel transition, and a high-grade SBO with two transition points (loops dilated up to 9 centimeters [cm] in diameter) (Image 1 and 2). In addition, there was engorgement of the superior mesenteric venous vasculature and associated mesenteric edema, suspicious for evolving bowel ischemia.

The patient was pan-cultured, decompressed with a nasogastric tube, and received empiric broad coverage for potential intra-abdominal infection with IV piperacillin-tazobactam. Norepinephrine and phenylephrine IV drips were started for pressor support in light of refractory hypotension. The colorectal surgery team was consulted, and the patient was taken for an emergent exploratory laparotomy. An estimated 75% of her small intestines was resected, followed by abdominal wall closure a few days later. Her postoperative course was uneventful. She was discharged home with close follow-up with colorectal surgery and gastroenterology. Biopsies taken during the resection displayed chronic active enteritis with strictures, transmural inflammation, non-necrotizing granulomas, and pyloric gland metaplasia, all of which are histologically compatible with active Crohn’s disease.

**CPC-EM Capsule**

What do we already know about this clinical entity?

*Inflammatory bowel disease flare-ups are common, with known complications of uncontrolled abdominal pain, abscess formation, or intestinal perforation.*

What makes this presentation of disease reportable?

*Midgut volvulus is extremely rare in adults, and its occurrence secondary to stricturing active Crohn’s disease is even more exceptional.*

What is the major learning point?

*When patients present with intermittent symptoms and inconclusive prior workup, it is imperative to consider atypical sequelae of the common diseases.*

How might this improve emergency medicine practice?

*This uncommon but dangerous complication of a prevalent pathology highlights the need to broaden the illness script related to heterogenous Crohn’s disease.*

## DISCUSSION

Midgut volvulus is more commonly seen in infants and children as the consequence of arrested normal rotation of the embryonic gut.^7^ In adult patients, intestinal malrotation is highly unusual but can occur secondary to adhesions after intra-abdominal surgeries, congenital defects, or malignancy.^8^ It is theorized that the simultaneous occurrence of small intestinal volvulus and Crohn’s disease is rare because the disease’s serosal inflammation causes adherence of the bowel to adjacent structures, making the bowel less likely to twist upon itself.^5^ Intestinal malrotation without the development of volvulus may present as chronic intermittent abdominal pain, nausea, and vomiting.^9^ This can be confirmed with an upper gastrointestinal (GI) series. The patient can undergo a non-emergent open or laparoscopic Ladd procedure to correct the abnormality.^9^ The Ladd procedure is an elective surgery in which malrotation is corrected to prevent a volvulus from occurring in the future. This procedure entails counterclockwise detorsion of the small intestine, surgical division of Ladd’s bands (naturally occurring fibrous stalks that attach the cecum to the right lower abdomen’s retroperitoneum), widening of the mesentery, appendectomy, and reorientation of the small intestine to the right and the colon to the left.^9^

In contrast, intestinal malrotation with volvulus is considered a life-threatening acute abdomen and warrants emergent surgery to salvage viable tissue. As in our case, patients often present acutely with complaints of severe abdominal pain, nausea, vomiting, hematochezia, or hematemesis, coupled with hemodynamic instability. However, chronic intestinal malrotation is a possibility. The clinical presentation of chronic malrotation often involves intermittent vomiting and abdominal pain, associated with food intolerance, malabsorption, and chronic diarrhea. Diagnosis of acute midgut volvulus can be confirmed via imaging, such as CT abdomen and pelvis or abdominal plain film. Patients should receive aggressive IV resuscitation and broad empiric antibiotics, due to the high risk of gut bacteria translocation, prior to being taken to the operating room for an emergent exploratory laparotomy. During laparotomy, the bowel is detorsed counterclockwise and resected if the tissue appears grossly necrotic.^10^ If the bowel is not necrotic-appearing but its viability is uncertain, it may be preserved and reinspected at a second-look operation scheduled approximately 24–48 hours later.^10^

The confirmation of Crohn’s disease can be diagnostically challenging as there is no definitive method and the heterogeneous disease can affect anywhere along the GI tract, including the rectum.^11^ Colonoscopy with biopsies is a common tool used by gastroenterologists for the initial evaluation of IBD.^12^ However, a standard colonoscopy can only reach up to the terminal ileum where the small intestine ends.^12^ Therefore, a normal colonoscopy does not exclude Crohn’s disease if the inflammation is located farther up along the small intestine. With recent advances in technology, video capsule endoscopy is becoming an increasingly popular method to aid in the diagnosis.^13^ In our case, the patient was, unfortunately, unable to engage in this expensive modality due to lack of insurance.

Even without a definitive diagnosis, emergency physicians should consider IBD in young and middle-aged patients with an ongoing history of intermittent abdominal pain. There can be an additional cognitive bias regarding individuals with functional labels, such as abdominal migraines or IBS.^14^ Serum inflammatory markers are often not reliable in IBD, as demonstrated in this scenario.^15^ Given the patient’s hemodynamic instability, severe metabolic derangements, and concerning physical exam, prompt recognition of her critically ill status and the pursuit of urgent imaging ultimately allowed her to have a favorable clinical outcome despite the extensive bowel resection that she required.

## CONCLUSION

Midgut volvulus is an extremely rare but dangerous sequela of Crohn’s disease, with only a handful of cases described in the literature. Prompt recognition of volvulus on imaging, rapid IV fluid resuscitation, and emergent exploratory laparotomy are the treatment. Our case highlights the dramatic consequences of undiagnosed Crohn’s disease in a patient with intermittent symptoms and extensive workup spanning over two decades.

## References

[1] Ballou S, Hirsch W, Singh P (2018). Emergency department utilisation for inflammatory bowel disease in the United States from 2006 to 2014. Aliment Pharmacol Ther.

[2] Jung YS, Park DI, Hong SN (2015). Predictors of urgent findings on abdominopelvic CT in patients with Crohn’s disease presenting to the emergency department. Dig Dis Sci.

[3] Miller G, Boman J, Shrier I (2000). Etiology of small bowel obstruction. Am J Surg.

[4] Kinjo F, Sunagawa T, Hokama A (2003). Crohn’s disease and volvulus. Gastrointest Endosc.

[5] Fink W, Cohen A, Greenstein AJ (1980). Small bowel volvulus in association with recurrent Crohn’s disease. Am J Gastroenterol.

[6] Fiorani C, Biancone L, Tema G (2014). Laparoscopic ileocolic resection for Crohn’s disease associated with midgut malrotation. JSLS.

[7] Kanellos-Becker I, Bergholz R, Reinshagen K (2014). Early prediction of complex midgut volvulus in neonates and infants. Pediatr Surg Int.

[8] Baty V, Rocca P, Fontaumard E (2002). Acute gastric volvulus related to adhesions after laparoscopic fundoplication. Surg Endosc.

[9] Kotobi H, Tan V, Lefevre J (2017). Total midgut volvulus in adults with intestinal malrotation. Report of eleven patients. J Visc Surg.

[10] Li X, Zhang J, Li B (2017). Diagnosis, treatment and prognosis of small bowel volvulus in adults: a monocentric summary of a rare small intestinal obstruction. PLoS One.

[11] Ha F, Khalil H (2015). Crohn’s disease: a clinical update. Therap Adv Gastroenterol.

[12] Cleynen I, Gonzalez JR, Figueroa C (2013). Genetic factors conferring an increased susceptibility to develop Crohn’s disease also influence disease phenotype: results from the IBDchip European Project. Gut.

[13] Yamada K, Nakamura M, Yamamura T (2021). Diagnostic yield of colon capsule endoscopy for Crohn’s disease lesions in the whole gastrointestinal tract. BMC Gastroenterol.

[14] Pines JM, Strong A (2019). Cognitive biases in emergency physicians: a pilot study. J Emerg Med.

[15] Schoepfer AM, Beglinger C, Straumann A (2010). Fecal calprotectin correlates more closely with the Simple Endoscopic Score for Crohn’s disease (SES-CD) than CRP, blood leukocytes, and the CDAI. Am J Gastroenterol.

